# Association Analysis of IL-17A and IL-17F Polymorphisms in Chinese Han Women with Breast Cancer

**DOI:** 10.1371/journal.pone.0034400

**Published:** 2012-03-26

**Authors:** Lihong Wang, Yongdong Jiang, Youxue Zhang, Yuwen Wang, Sunhui Huang, Zhihua Wang, Baoling Tian, Yue Yang, Wei Jiang, Da Pang

**Affiliations:** 1 Institute of Cancer Prevention and Treatment, Harbin Medical University, Harbin, China; 2 Department of Breast Surgery, The Third Affiliated Hospital of Harbin Medical University, Harbin, China; 3 Department of Laboratory Diagnostics, The Second Affiliated Hospital of Harbin Medical University, Harbin, China; 4 College of Bioinformatics Science and Technology, Harbin Medical University, Harbin, China; IPO, Inst Port Oncology, Portugal

## Abstract

**Background:**

Research into the etiology of breast cancer has recently focused on the role of the immunity and inflammation. The proinflammatory cytokines IL-17A and IL-17F can mediate inflammation and cancer. To evaluate the influences of IL-17A and IL-17F gene polymorphisms on the risk of sporadic breast cancer, a case-control study was conducted in Chinese Han women.

**Methodology and Principal Findings:**

We genotyped three single-nucleotide polymorphisms (SNPs) in IL-17A (rs2275913, rs3819025 and rs3748067) and five SNPs in IL-17F (rs7771511, rs9382084, rs12203582, rs1266828 and rs763780) to determine the haplotypes in 491 women with breast cancer and 502 healthy individuals. The genotypes were determined using the SNaPshot technique. The differences in the genotypic distribution between breast cancer patients and healthy controls were analyzed with the Chi-square test for trends. For rs2275913 in IL-17A, the frequency of the AA genotype was higher in patients than controls (*P* = 0.0016). The clinical features analysis demonstrated significant associations between IL-17 SNPs and tumor protein 53 (P53), progesterone receptor (PR), human epidermal growth factor receptor 2 (Her-2) and triple-negative (ER-/PR-/Her-2-) status. In addition, the haplotype analysis indicated that the frequency of the haplotype A_rs2275913_G_rs3819025_G_rs3748067_, located in the IL-17A linkage disequilibrium (LD) block, was higher in patients than in controls (*P* = 0.0471 after correction for multiple testing).

**Conclusions and Significance:**

Our results suggested that SNPs in IL-17A but not IL-17F were associated with the risk of breast cancer. Both IL-17A and IL-17F gene polymorphisms may provide valuable information for predicting the prognosis of breast cancer in Chinese women.

## Introduction

Breast cancer is the most common malignancy in women worldwide and its rate is increasing in both developed and developing countries [1]. The etiology of breast cancer is complicated and not completely understood, but recent studies have focused on the roles of immunity and inflammation [2]. Th17 cells have recently been identified as a new subset of T helper cells, in addition to the traditional Th1 and Th2 subsets. Th17 cells are generally important mediators of inflammation [3], autoimmune diseases [4] and cancer [5], [6], particularly through the production of IL-17A and IL-17F.

IL-17 is a novel family of proinflammatory cytokines that consists of six similar members, designated IL-17A (also previously known as IL-17), IL-17B, IL-17C, IL-17D, IL-17E and IL-17F, according to the order in which they were discovered [7]. IL-17A and IL-17F lie immediately adjacent to one another on human chromosome 6, and both cytokines are produced by Th17 cells in response to IL-23 [8], [9]. Several studies have found overexpressing of IL-17A in various tumor tissues, including multiple myeloma, ovarian cancers, gastric cancer and breast cancer [10]–[13].

Kawaguchi et al have shown that the IL-17F 7488T/C (rs763780) variant, which causes a His-to-Arg substitution at amino acid 161 (H161R), antagonizes the function of wild-type IL-17F and influences the risk of asthma [14]. Polymorphisms in IL-17A (rs2275913) and IL-17F (rs763780) have recently been identified to be associated with the susceptibility to rheumatoid arthritis, gastric cancer and ulcerative colitis, respectively [15]–[17]. However, it remains unknown whether genetic polymorphisms in IL-17A and IL-17F influence the risk of breast cancer development.

To investigate the role of the inflammatory cytokines IL-17A and IL-17F in the pathogenesis of breast cancer, we selected tagged single-nucleotide polymorphisms (SNPs) in the IL-17A and IL-17F genes with Haploview software and then evaluated the genotypes of these SNPs in breast cancer patients and controls from the Chinese Han population. We also determined the relationship between the polymorphisms in the IL-17A and IL-17F genes and several prognostic factors, including tumor protein 53 (P53), estrogen receptor (ER), progesterone receptor (PR), human epidermal growth factor receptor-2 (Her-2) and triple-negative breast cancer (TNBC) in breast cancer patients.

## Results

### Genotypic and allelic frequencies

The genotypic and allelic frequencies of the IL-17A and IL-17F polymorphisms in breast cancer patients and healthy controls are shown in Table 1 and Table 2. All eight of the SNPs genotyped were in Hardy-Weinberg equilibrium (HWE) (*P*>0.05) in our case and control cohorts. Only the frequencies of the AA genotype and the A allele in rs2275913, located at position −197 from the starting codon of the IL-17A gene, were statistical significant higher in breast cancer patients than in controls (*P* = 0.0016 and *P* = 0.0029, respectively). After highly conservative correction for multiple testing, a significant association was also observed for the A allele of rs2275913 (*P* = 0.0311). The other seven SNPs (rs3819025 and rs3748067 in the IL-17A gene and rs7771511, rs9382084, rs12203582, rs1266828 and rs763780 in the IL-17F gene) showed no significant differences in genotypic distribution between the cases and the controls (*P*>0.05).

**Table 1:** Genotypes of eight SNPs in the IL-17A and IL-17F genes.

Reference SNP ID	Genotype	Frequency no. (%)	*P* value	Odds ratio (95% CI)	
		Patients (*n* = 491)	Controls (*n* = 502)		
rs2275913†	G/G	165 (33.60)	198 (39.52)	0.1973	
	A/G	234 (47.66)	245 (48.90)	0.6950	
	A/A	92 (18.74)	58 (11.58)	0.0016	1.76 (1.24–2.51)
rs3819025†	G/G	342 (69.65)	344 (68.66)	0.7354	
	A/G	132 (26.88)	145 (28.94)	0.4700	
	A/A	17 (3.46)	12 (2.40)	0.3185	
rs3748067‡	G/G	336 (68.43)	332 (66.53)	0.5237	
	A/G	133 (27.09)	142 (28.46)	0.6306	
	A/A	22 (4.48)	25 (5.01)	0.6953	
rs763780	T/T	382 (77.80)	396 (78.89)	0.6784	
	C/T	103 (20.98)	99 (19.72)	0.6229	
	C/C	6 (1.22)	7 (1.39)	0.8111	
rs7771511	C/C	385 (78.41)	396 (78.88)	0.8556	
	C/T	100 (20.37)	100 (19.92)	0.8608	
	T/T	6 (1.22)	6 (1.20)	0.9692	
rs12203582†	G/G	201 (40.94)	226 (45.11)	0.7555	
	A/G	223 (45.42)	210 (41.92)	0.2663	
	A/A	67 (13.65)	65 (12.97)	0.1845	
rs9382084	T/T	209 (42.57)	207 (41.24)	0.1673	
	G/T	226 (46.03)	223 (44.42)	0.6111	
	G/G	56 (11.41)	72 (14.34)	0.6708	
rs1266828	T/T	341 (69.45)	345 (68.73)	0.9492	
	C/T	134 (27.29)	141 (28.09)	0.7589	
	C/C	16 (3.26)	16 (3.19)	0.8048	

† controls = 501, missing = 1;

‡ controls = 499, missing = 3.

**Table 2:** Allele frequencies of eight SNPs in the IL-17A and IL-17F genes.

Reference SNP ID	Allele	Frequency no. (%)	*P* value	Odds ratio (95% CI)	
		Patients (*n* = 491)	Controls (*n* = 502)		
rs2275913†	A	418 (42.57)	361 (36.03)	0.0029^a^	1.32 (1.10–1.58)
A/G	G	564 (57.43)	641 (63.97)		
rs3819025†	G	816 (83.10)	833 (83.13)	0.9820	
A/G	A	166 (16.90)	169 (16.87)		
rs3748067‡	G	805 (81.98)	806 (80.76)	0.4879	
A/G	A	177 (18.02)	192 (19.24)		
rs763780	T	867 (88.29)	891 (88.75)	0.7500	
C/T	C	115 (11.71)	113 (11.25)		
rs7771511	C	870 (88.59)	892 (88.85)	0.8603	
C/T	T	112 (11.41)	112 (11.15)		
rs12203582†	G	625 (63.65)	662 (66.07)	0.2585	
A/G	A	357 (36.35)	340 (33.93)		
rs9382084	T	644 (65.58)	637 (63.45)	0.3203	
G/T	G	338 (34.42)	367 (36.55)		
rs1266828	T	816 (83.10)	831 (82.77)	0.8465	
C/T	C	166 (16.90)	173 (17.23)		

† controls = 501, missing = 1;

‡ controls = 499, missing = 3.

a *P* = 0.0311 after correcting the *P*-value for multiple testing with the Haploview program using 10,000 permutations.

### Haplotypes analysis

The associations of IL-17A and IL-17F SNPs with breast cancer were further analyzed with the Haploview program. Two blocks were identified with the software as shown in Figure S1. In the IL-17A block, the frequency of the haplotype A_rs2275913_G_rs3819025_G_rs3748067_ was higher in patients than in controls (*P* = 0.0045), whereas the frequency of the haplotype G_rs2275913_G_rs3819025_G_rs3748067_ was lower (*P* = 0.0285) in patients than in controls. However, after the *P*-value was corrected for multiple testing using the permutation option in the Haploview software, a significant association was only found for haplotype A_rs2275913_G_rs3819025_G_rs3748067_ (*P* = 0.0471). In the IL-17F block, we did not find any haplotypes that were associated with breast cancer (Table 3).

**Table 3:** Haplotype frequencies in breast cancer patients and healthy controls.

Block	Haplotype	Freq.	Breast Cancer (*n* = 491)	Healthy Controls (*n* = 502)	*P* value
IL-17A	AGG	0.391	0.422	0.360	0.0045^a^
	GGG	0.254	0.232	0.275	0.0285
	GGA	0.184	0.173	0.195	0.2051
	GAG	0.167	0.165	0.169	0.8338
IL-17F	TCATT	0.349	0.362	0.337	0.2383
	TCGGT	0.185	0.176	0.194	0.2991
	TCGTT	0.181	0.178	0.184	0.7103
	TCGGC	0.171	0.168	0.173	0.7997
	CTGTT	0.113	0.114	0.111	0.8049

The haplotypes contain the following SNPs in the order listed: IL-17A: rs2275913, rs3819025 and rs3748067; IL-17F: rs763780, rs7771511, rs12203582, rs9382084 and rs1266828. The haplotypes occurred with a frequency of = 10% in the case-control population.

a *P* = 0.0471 after correcting the *P*-value for multiple testing with the Haploview program using 10,000 permutations.

### Clinical features

We analyzed the associations between the polymorphisms in the IL-17A and IL-17F genes and a series of clinicopathologic features, including clinical stage, lymph node metastasis, tumor size, and the statuses of P53, ER, PR, Her-2 and TNBC. We found no significant associations between IL-17A and IL-17F SNPs and clinical stage, lymph node metastasis or ER status (*P*>0.05).

As shown in Table 4, the frequency of genotype GG in rs2275913 was higher in P53-positive cases (*P* = 0.0302), whereas the frequency of the G allele in rs3748067 was lower (*P* = 0.0357) in P53-positive cases. The polymorphisms in rs2275913, rs3748067 and rs9382084 showed statistical differences in PR status (*P*<0.05). The frequencies of genotype AA in rs2275913 (*P* = 0.0350) and genotype GG in rs3748067 (*P* = 0.0312) were lower in PR-positive cases, whereas the frequencies of genotype AG in rs3748067 (*P* = 0.0146) and genotype GG in rs9382084 (*P* = 0.0295) were higher in PR-positive cases. The frequency of genotype AG in rs3819025 was higher (*P* = 0.0236) and the frequency of genotype AA in rs12203582 was lower (*P* = 0.0258) in Her-2-positive cases. Finally, our findings indicated that the frequency of genotype AA in rs3748067 was higher (*P* = 0.0243) in patients with TNBC, and the frequency of the G allele was higher in patients with tumors = 2 cm (*P*<0.0001).

**Table 4:** Clinical features associated with IL-17A and IL-17F gene polymorphisms.

Clinical feature	Allele and genotype of SNP	Frequency no. (%)	*P* value	Odds ratio (95% CI)		
		Positive	Negative			
P53	rs2275913	GG	41 (41.84)	101 (30.15)	0.0302	0.60 (0.38–0.95)
	rs3748067	G	150 (76.53)	557 (83.13)	0.0357	0.66 (0.45–0.97)
PR	rs2275913	AA	35 (2.86)	45 (22.61)	0.0350	1.69 (1.04–2.74)
	rs3748067	AG	76 (32.07)	43 (21.61)	0.0146	0.58 (0.38–0.90)
	rs3748067	GG	151 (63.71)	146 (73.37)	0.0312	1.57 (1.04–2.36)
	rs9382084	GG	35 (14.77)	16 (8.04)	0.0295	0.50 (0.93–0.27)
Her-2	rs3819025	AG	42 (34.71)	75 (23.96)	0.0236	0.59 (0.38–0.93)
	rs12203582	AA	10 (8.26)	52 (16.61)	0.0258	2.21 (1.10–4.44)
TNBC	rs3748067	AA	7 (9.59)	15 (3.59)	0.0243	2.85 (1.16–6.99)

We also analyzed the associations between each haplotypes and the clinical features of the cases as predictive factors. Haplotype G_rs2275913_G_rs3819025_A_rs3748067_ of IL-17A had a lower frequency in P53-positive cases (*P* = 0.0275), and haplotype T_rs763780_C_rs7771511_G_rs12203582_G_rs9382084_T_rs1266828_ of IL-17F had a lower frequency in PR-positive cases (*P* = 0.0289) (Table 5).

**Table 5:** Clinical features associated with haplotypes.

Clinical feature	Block	Haplotype	Freq.	Positive	Negative	*P* value
P53	IL-17A	GGA	0.175	0.159	0.227	0.0275
PR	IL-17F	TCGGT	0.175	0.144	0.200	0.0289

The haplotypes contain the following SNPs in the order listed: IL-17A: rs2275913, rs3819025 and rs3748067; IL-17F: rs763780, rs7771511, rs12203582, rs9382084 and rs1266828. The haplotypes shown occurred with a frequency of = 10% in the case-control population.

## Discussion

Numerous data have shown that chronic inflammation is associated with breast cancer [18], [19]. Th17 cells mediate chronic inflammation [3] and cancer [5], [20]. IL-17A and IL-17F cytokines are both expressed by Th17 cells, and they play a role in coordinating local tissue inflammation by inducing the release of proinflammatory and neutrophil-mobilizing cytokines. Although the significance of IL-17A and IL-17F in the pathogenesis of breast cancer is unclear, we hypothesized that both cytokines may affect the development of breast cancer, either coordinately or independently. Therefore, we investigated the influence of the polymorphisms in these two cytokines in the pathogenesis of breast cancer.

To date, no studies have investigated the association between IL-17 gene polymorphisms and human breast cancer. In our case-control study, for the first time we investigated the associations between eight SNPs in the IL-17A (rs2275913, rs3819025 and rs3748067) and IL-17F (rs763780, rs7771511, rs12203582, rs9382084 and rs1266828) genes and the risk of sporadic breast cancer in Chinese Han women. Genotyping of the eight SNPs in our study showed that only rs2275913, located at position −197 from the starting codon of the IL-17A gene, was correlated with the risk of breast cancer. The homozygous AA genotype and the A allele were more frequent in breast cancer patients. In the IL-17A block, the frequency of haplotype A_rs2275913_G_rs3819025_G_rs3748067_ was higher in patients than in the controls. The other seven SNPs showed no statistical significant differences in genotypic distribution between breast cancer cases and the controls. Therefore, our investigation suggests that IL-17A, but not IL-17F, contributes to breast cancer susceptibility, and the Chinese women with these risk factors in the IL-17A gene may have an increased susceptibility to breast cancers.

From the analysis of clinical information in conjunction with IL-17A or IL-17F gene polymorphisms, we found that the IL-17A and IL-17F genes may influence the diagnosis, treatment, and prognosis of breast cancer. In the P53 analysis, we found that the frequency of the rs2275913 GG genotype was higher and the frequency of the rs3748067 G allele was lower in P53-positive cases. The suppressor gene P53 is important in the treatment and prognosis of breast cancer and the mutations in P53 can lead to tumor metastasis and poor prognosis [21]. Both of these two SNPs are located in the IL-17A gene, which indicates that IL-17A polymorphisms are associated with P53 status and the occurrence and progression of breast cancer.

The frequencies of the rs2275913 AA and rs3748067 GG genotypes in IL-17A were lower in PR-positive patients, whereas the frequency of the rs9382084 GG genotype in IL-17F was higher in PR-positive patients. These SNPs in IL-17A and IL-17F were significantly associated with PR status. Steroid hormone receptors influence the disease-free and overall survival of breast cancer patients and are considered to be predictive markers of endocrine therapy [22], [23]. Thus, the rs2275913 AA and rs3748067 GG genotypes may be factors of poor prognosis and ineffective treatment, whereas the rs9382084 GG genotype may be a good marker of breast cancer. We also found that the frequencies of rs3819025 AG in IL-17A and rs12203582 AA in IL-17F were lower in Her-2-positive patients than in Her-2-negative patients. Hormone and Her-2 receptors are two important pharmaceutical targets that affect the survival of patients with metastatic breast cancer [24]. In addition, the humanized antibody targeting Her-2 (Trastuzumab) is used in combination with chemotherapy to treat patients with breast cancers overexpressing Her-2. These data indicate that patients with the rs3819025 AG genotype or the rs12203582 AA genotype may be insensitive to Trastuzumab. According to these results, the variants of IL-17A and IL-17F may be important in the prognosis of breast cancer and determining the effectiveness of pharmaceuticals treatments.

Furthermore, our findings also indicated that the frequency of the rs3748067 AA genotype in the 3′UTR of IL-17A was higher in patients with TNBC, which suggested that patients homozygous for AA in rs3748067 may have a poor prognosis. TNBC is defined by the absence of ER, PR and Her-2 expression, as assessed through immunohistochemical analysis. Because they lack these common therapeutic targets, patients with TNBC have a worse prognosis and tends to relapse early compared with patients with other subtypes of breast cancer [25]. The 3′UTR can regulate gene expression at different levels and mediate the stability, degradation, and subcellular localization of mRNA [26]. Additionally, we found that the frequency of the G allele of rs3748067 was higher in patients with tumors = 2 cm. Therefore, the polymorphism in rs3748067 may change the IL-17A expression and further affect the prognosis of breast cancer patients.

Finally, the results of our haplotype analysis demonstrated that the haplotype G_rs2275913_G_rs3819025_A_rs3748067_ of IL-17A and the haplotype T_rs763780_C_rs7771511_G_rs12203582_G_rs9382084_T_rs1266828_ of IL-17F were less frequent in patients with P53- and PR-positive statuses, respectively. Therefore, haplotype G_rs2275913_G_rs3819025_A_rs3748067_ may be a marker of good prognosis, whereas haplotype T_rs763780_C_rs7771511_G_rs12203582_G_rs9382084_T_rs1266828_ may be a marker of poor prognosis for breast cancer patients.

In conclusion, our study determined the relationships between IL-17A and IL-17F gene polymorphisms and the risk of breast cancer in Chinese women. These results suggested that SNPs in IL-17A but not IL-17F had a significant association with breast cancer risk. Both IL-17A and IL-17F gene polymorphisms may provide valuable information for predicting the prognosis and effectiveness of pharmaceutical treatment in Chinese breast cancer patients.

## Materials and Methods

### Subjects

Our study included 491 Chinese women with breast cancer but no family history of cancer and 502 healthy women. The sporadic breast cancer patients were selected from the Department of Breast Surgery (The Third Affiliated Hospital of Harbin Medical University, Heilongjiang Province) and ranged in age from 27 to 91 years old (mean age 49.5±10.3 years). The breast cancer patients were diagnosed based on surgical and pathological symptoms. The control group consisted of 502 age-matched healthy Han women (mean age 49.0±9.9 years) who were volunteers in the same district and had no history of tumors or inflammatory diseases. And imaging examinations were done for the volunteers to rule out breast cancer. Both the breast cancer group and the healthy control group were from the Heilongjiang Province of China and all of the blood samples were obtained from September 2008 to May 2009. The Ethics Committee of Harbin Medical University approved the study protocol, and written informed consent was obtained from all of the participating subjects.

### Clinicopathological evaluation

The clinicopathologic information of breast cancer patients was shown in Table 6. We retrospectively evaluated conventional clinicopathological factors and the immunohistochemistry (IHC) results for four biological factors (ER, ZM0104; PR, ZM0215; Her-2, ZM0065; P53, ZA-0501; Beijing ZhongShan GoldenBridge Biological Technology CO., LTD, China) using paraffin embedded tissues according to the reported recommendations for tumor marker prognostic studies (REMARK) [27]. The tumor clinical stage was assessed according to the criteria described in the International Union Against Cancer (UICC).

**Table 6:** Clinicopathologic description of the breast cancer patients (*n* = 491).

Clinicopathologic information	Case no. (%)
Histology	
Infiltrative ductal carcinoma	420 (85.54)
Intraductal carcinoma	36 (7.33)
Mucinous adenocarcinoma	13 (2.65)
Infiltrative lobular carcinoma	12 (2.44)
Medullary carcinoma	4 (0.81)
Others	6 (1.22)
Clinical Stage (UICC)	
0	2 (0.41)
I	124 (25.25)
II	256 (52.14)
III	37 (7.54)
IV	8 (1.63)
Unknown	64 (13.03)
Tumor size (cm)	
TZ = 2	236 (48.07)
2<TZ = 5	223 (45.42)
TZ>5	28 (5.70)
Unknown	4 (0.81)
LN involvement	
Positive	204 (41.55)
Negative	261 (53.15)
Unknown	26 (5.30)
P53	
Positive	98 (19.96)
Negative	335 (68.23)
Unknown	58 (11.81)
ER	
Positive	266 (54.18)
Negative	169 (34.42)
Unknown	56 (11.40)
PR	
Positive	237 (48.27)
Negative	199 (40.53)
Unknown	55 (11.20)
Her-2	
Positive	121 (24.64)
Negative	313 (63.75)
Unknown	57 (11.61)
IHC subtype	
ER or PR(+)/Her-2(−)	240 (48.88)
ER or PR(+)/Her-2(+)	52 (10.59)
ER(−)/PR(−)/Her-2(−)	73 (14.87)
ER(−)/PR(−)/Her-2(+)	69 (14.05)
Unknown	57 (11.61)

**Abbreviations:**

LN, lymph node; P53, tumor protein 53; ER, estrogen receptor; PR, progesterone receptor; TZ, tumor size; Her-2, human epidermal growth factor receptor-2; IHC, immunohistochemistry.

A cut-off value of 10% of positively stained nuclei was used to define ER and PR positivity; Her-2 was scored as 0–3+ according to membrane staining. Cases with IHC scores of 3+ or 2+ with gene amplification by fluorescence in situ hybridization (FISH) were considered positive for Her-2. Cells with positive staining for P53 were counted and expressed as a percentage. We scored the immunostained slides as 0–3+ (0, negative; 1+,  = 25%; 2+, 25–50%; 3+, >50%). For the prognosis comparison, low expression was defined as P53 = 25% (median values for all evaluated tumors).

### DNA extraction

Genomic DNA was isolated from EDTA anti-coagulated whole blood using the AxyPrep Blood Genomic DNA Miniprep Kit (Axygen Biotechnology, USA). Each DNA sample was stored at −20°C until analysis.

### SNP selection and genotyping

SNP selection performed with Haploview v4.0 through pairwise tagging (*r* ^2^>0.80) and minor allele frequencies >0.1 using Chinese (CHB) genotype data from HapMap (http://www.hapmap.org/) covering the IL-17A and IL-17F genes. Two SNPs (rs3819025 and rs3748067) of the IL-17A gene and four SNPs (rs7771511, rs9382084, rs12203582 and rs1266828) of the IL-17F gene were selected as tags. The SNP rs2275913 in the IL-17A gene promoter and the non-synonymous coding SNP rs763780 (p.His161Arg) in the IL-17F gene were also selected because they are associated with some cancers.

The SNaPshot SNP assay was performed to detect the dimorphism at the eight SNP loci. The PCR primer pairs used to amplify the DNA sequences are shown in Table S1. PCR was performed in a 20 µl reaction mixture containing 1 µl (10 ng) of template DNA, 1 µM of each primer, 0.3 mM of each deoxynucleotide triphosphate, 3.0 mM of MgCl_2_, and 1 U HotStarTaq polymerase (Qiagen Inc., USA) with 1× HotStarTaq buffer. The PCR program was consisted of an initial melting step of 15 minutes at 95°C; 11 cycles of 20 seconds at 94°C, 40 seconds at 65°C-0.5°C/cycle, and 90 seconds at 72°C; 24 cycles of 20 seconds at 94°C, 30 seconds at 59°C, and 90 seconds at 72°C; and a final elongation step of 2 minutes at 72°C. To purify the PCR products, 1 U SAP and 1 U Exonuclease I were mixed with 10 µl PCR product for 1 hour at 37°C and 15 minutes at 75°C. The SNaPshot multiplex single-base extension reaction primer sequences are shown in Table S1. The extension reaction was performed in a 10 µl reaction mixture containing 5 µl of the SNaPshot Multiplex Kit (Applied Biosystems, USA), 2 µl of purified PCR products, 0.8 µM of the extension reaction primer, and 2 µl water. The PCR program was consisted of 1 minute at 96°C; 28 cycles of 10 seconds at 96°C, 5 seconds at 50°C, and 30 seconds at 60°C; and 4°C as the holding temperature. Finally, 10 µl of the extension product was purified with 1 U SAP for 1 hour at 37°C and inactivated for 15 minutes at 75°C.

The resulting data were analyzed with an ABI3130XL sequencer and GeneMapper™ 4.0 Software (Applied Biosystems, Co. Ltd., USA). The SNP genotyping figure is shown in Table S2. To ensure quality control (QC), genotyping was performed by researchers blinded to the case/control status of the subjects, and a random sample of 5% of the cases and controls was genotyped twice by different persons, with a reproducibility of 100%.

### Statistical analysis

The genotypes in both data sets were in HWE. The frequencies of alleles and genotypes were obtained through direct counting. The statistical significance was defined as *P*<0.05. We used Haploview software to construct haplotypes and estimate haplotype frequencies for both cases and controls. Only the haplotypes that occurred at a frequency of = 10% in the case-control population were selected in our study. To obtain a measure of significance that was corrected for multiple testing bias, we ran 10,000 permutations to compute *P*-values using the Haploview program. The comparisons of the distributions of the genotype, allele and haplotype frequencies were performed using the Chi-square test. The relative risk associated with rare alleles, genotypes and haplotypes was estimated as an odds ratio (OR) with a 95% confidence interval (CI).

## Supporting Information

Figure S1 **Linkage disequilibrium (LD) block defined with the Haploview program based on the solid spine of LD method. Pairwise LD coefficients D′×100 are shown in each cell.** The standard color scheme was applied for the LD color display.
(TIF)

Table S1 **Product size and primer sequences of eight SNPs in the IL-17A and IL-17F genes.**
(DOC)

Table S2 **SNP genotyping figure.**
(PDF)
