# Supplementary figures and images for: Association Analysis of IL-17A and IL-17F Polymorphisms in Chinese Han Women with Breast Cancer
Source: PLoS One. 2012 Mar 26;7(3):e34400. doi: 10.1371/journal.pone.0034400 (PMC3312906; doi:10.1371/journal.pone.0034400)

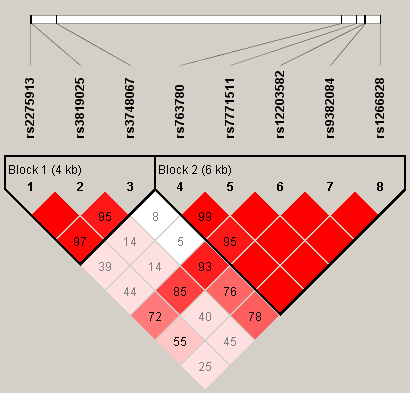

Supplement: Figure S1 — Linkage disequilibrium (LD) block defined with the Haploview program based on the solid spine of LD method. Pairwise LD coefficients D′×100 are shown in each cell. The standard color scheme was applied for the LD color display. (TIF) [file pone.0034400.s001.tif]

Table S2 SNP genotyping figure

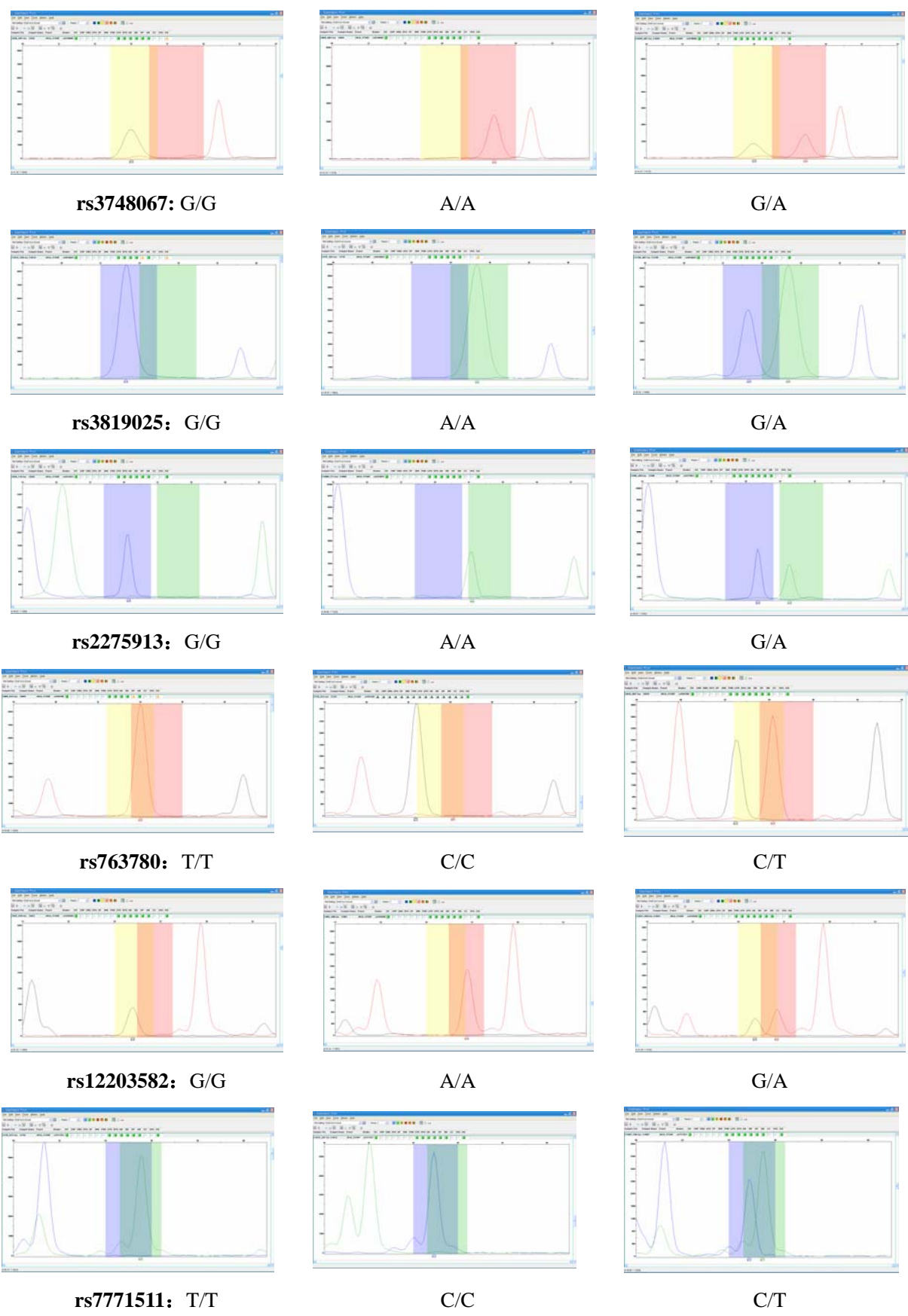

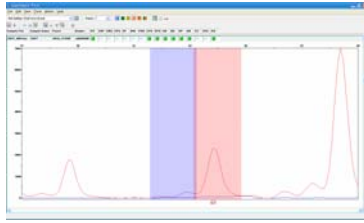

**rs9382084: T/T**

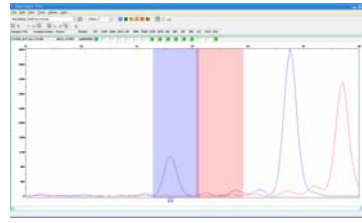

**G/G**

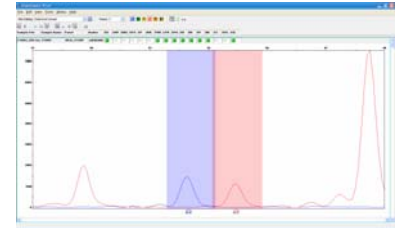

**G/T**

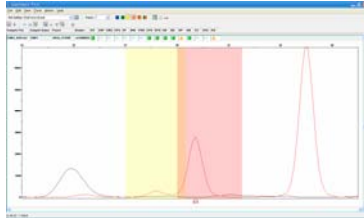

**rs1266828: T/T**

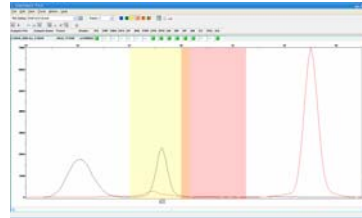

**C/C**

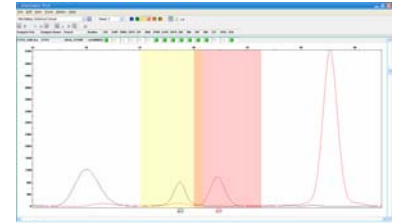

**C/T**

Supplement: Table S2 — SNP genotyping figure. (PDF) [file pone.0034400.s003.pdf]
