# Supplementary material for: Association Analysis of IL-17A and IL-17F Polymorphisms in Chinese Han Women with Breast Cancer
Source: PLoS One. 2012 Mar 26;7(3):e34400. doi: 10.1371/journal.pone.0034400 (PMC3312906; doi:10.1371/journal.pone.0034400)
Supplement: Table S1 — Product size and primer sequences of eight SNPs in the IL-17A and IL-17F genes. (DOC) [file pone.0034400.s002.doc]

**Table S1** Product size and primer sequences of eight SNPs in IL-17A and IL-17F genes

| Reference SNP ID | Product Size (bp) | PCR primer sequence | Extension primer sequence |
| --- | --- | --- | --- |
| rs2275913 | 210 | F:GCCCTTCCCATTTTCCTTCAGA  R:CCAATCAACTGGGGATGGATGA | SF:TTTTTTTTTTCCTTCCCATTTTCCTTCAGAAG |
| rs3819025 | 246 | F:GGTGTCACCCCTGAACCCACT  R:CATGCCCACGGTCCAGAAATA | SF:TTGGTGGTGAGTCCTGCACTAAC |
| rs3748067 | 217 | F:AAGCAGGGAGCCTGCAGAGTG  R:GGCACCACACAACCCAGAAAG | SR:GCTGATGGGGCAGAACGAA |
| rs763780 | 188 | F:CTGTTTCCATCCGTGCAGGTC  R:TGGTGACTGTTGGCTGCACCT | SF:TTTTTTTTTTTTTGGATATGCACCTCTTACTGCACA |
| rs7771511 | 227 | F:CCTATCCTGGCTTCCTTGCTGAG  R:GTAGAGGGGTGGCTCCGAAGA | SR:TTTTTTTTTTTTTTTTTTTTTTTTCAAGTTGGAAATAAAAACAAGAGCTATC |
| rs12203582 | 234 | F:TCATCAACTTTCATCCCCCACAA  R:TTGGATACTTATCATATGCTGCCAAGTG | SR:TTTTTTTTTTTTTTTTTTTTTTTTCAAGAGCTAGTTGTTGAATGAATGAA |
| rs9382084 | 186 | F:TCAGGGCATGGGCTTCAGAGT  R:TTAGCAAGAGTCGCCAGTATTGGAT | SF:TTTTTTTTTTTTTTTTTTTTTTTTTTTTTTTTTAACTCAATTCCAGGACTCTTAGAAGC |
| rs1266828 | 199 | F:CCCAGCAGGTCTGGAATCTTG  R:CCATTGCTATATGCCATGAACCTTTTA | SF:TTTTTTTTTGAAAACAGGGGTTAGGAAATCC |
